# Characterization of a Putative Receptor Binding Surface on Skint-1, a Critical Determinant of Dendritic Epidermal T Cell Selection*[Fn FN2]

**DOI:** 10.1074/jbc.M116.722066

**Published:** 2016-02-25

**Authors:** Mahboob Salim, Timothy J. Knowles, Rosie Hart, Fiyaz Mohammed, Martin J. Woodward, Carrie R. Willcox, Michael Overduin, Adrian C. Hayday, Benjamin E. Willcox

**Affiliations:** From the ‡ Cancer Immunology and Immunotherapy Centre, Institute of Immunology and Immunotherapy, University of Birmingham, Edgbaston, Birmingham B15 2TT,; the § School of Biosciences, University of Birmingham, Edgbaston, Birmingham B15 2TT,; the ¶ Francis Crick Institute, Lincoln's Inn Fields Research Laboratories, London WC2A 3LY,; the ** School of Cancer Sciences, University of Birmingham, Henry Wellcome Building for Biomolecular NMR, Edgbaston, Birmingham B15 2TT, and; the ‖ Peter Gorer Department of Immunobiology, King's College London, London SE1 9RT, United Kingdom

**Keywords:** immunology, lymphocyte, nuclear magnetic resonance (NMR), stress, T-cell receptor (TCR)

## Abstract

Dendritic epidermal T cells (DETC) form a skin-resident γδ T cell population that makes key contributions to cutaneous immune stress surveillance, including non-redundant contributions to protection from cutaneous carcinogens. How DETC become uniquely associated with the epidermis was in large part solved by the identification of *Skint-1*, the prototypic member of a novel B7-related multigene family. Expressed only by thymic epithelial cells and epidermal keratinocytes, Skint-1 drives specifically the development of DETC progenitors, making it the first clear candidate for a selecting ligand for non-MHC/CD1-restricted T cells. However, the molecular mechanisms underpinning Skint-1 activity are unresolved. Here, we provide evidence that DETC selection requires Skint-1 expression on the surface of thymic epithelial cells, and depends upon specific residues on the CDR3-like loop within the membrane-distal variable domain of Skint-1 (Skint-1 DV). Nuclear magnetic resonance of Skint-1 DV revealed a core tertiary structure conserved across the Skint family, but a highly distinct surface charge distribution, possibly explaining its unique function. Crucially, the CDR3-like loop formed an electrostatically distinct surface, featuring key charged and hydrophobic solvent-exposed residues, at the membrane-distal tip of DV. These results provide the first structural insights into the Skint family, identifying a putative receptor binding surface that directly implicates Skint-1 in receptor-ligand interactions crucial for DETC selection.

## Introduction

Dendritic epidermal T cells (DETC)^5^ form a unique skin-resident γδ T cell population that makes key non-redundant contributions to cutaneous immune stress surveillance (1), including profound protection from carcinogenesis (2–4). In the skin, DETC appear to be activated by signals from adjacent damaged or stressed keratinocytes (5), including ligands for the activating receptor NKG2D expressed by DETCs, as well as signals through the T cell receptor (TCR). Once activated, DETCs are involved in regulating inflammation, modulating downstream immune responses, and maintaining epithelial integrity, via a combination of cytolysis and chemokine and cytokine production (6–8).

The TCR repertoire of DETC is strikingly oligoclonal/pseudo-monoclonal comprising Vγ5Vδ1 chains with germline-encoded junctional regions (9). This observation pre-empted subsequent discoveries of other cells with limited TCR repertoires, *e.g.* NKT cells, and raised the question as to whether such TCRs are conventionally responsive to challenge, or primarily mediate other forms of interaction, *e.g.* with their host tissue. Consistent with this, the DETC TCR constitutively transmits partial signals from focal points of contact with keratinocytes that are disrupted by stress (10). This unusual phenotype, relative to systemic T cells, is consistent with altered antigen-receptor responses of innate-like T cells that can also be strongly activated independent of TCR activation (11).

As the first T cells to be produced by the fetal murine thymus, DETC provide almost unique insight into the development of non-MHC/CD1-restricted T cells. Evidence that the cells' TCR uniformity is in large part a product of positive selection was long-standing (12–14), and seemingly cemented by the identification of the *Skint-1* gene as critical for thymic selection of DETCs (15, 16). *Skint-1* mRNA is only expressed by thymic epithelial cells and keratinocytes (15), consistent with it directly mediating thymic selection of DETC progenitors and thereafter regulating their homing to the epidermis (15, 17, 18).

Nonetheless, the molecular and structural basis of Skint-1 function is unclear. *Skint-1* encodes a 364-amino acid protein of unusual topology, containing two Ig-like domains (one IgV-like and one IgC-like domain), but also three transmembrane domains and a short C-terminal cytoplasmic tail (supplemental Fig. S1). It is the prototypic member of an uncharacterized family of B7-like molecules also including *Skint-2* to -*11*, which despite exhibiting significant similarity to *Skint-1*, cannot compensate for Skint-1 in mediating DETC selection. Previous experiments have highlighted multiple regions of Skint-1 as being important for DETC selection, including the membrane-distal immunoglobulin variable domain of the molecule (Skint-1 DV) (17), but did not shed light on the underlying mechanisms.

Here, we used thymic organ culture, mutagenesis, and structural biology approaches to probe the molecular basis of Skint-1 function, focusing on the structure and function of Skint-1 DV. We show that DETC selection depends upon cell-surface expression of Skint-1, and upon specific residues within a region equivalent to the CDR3 loop of immunoglobulins that sits within an exposed surface of the membrane-distal region. These data argue that Skint-1-mediated selection results from its direct involvement in cell surface receptor-ligand interactions.

## Experimental Procedures

### 

#### 

##### Antibody Generation

Several monoclonal antibodies specific for Skint-1 DV were generated from rat serum following immunization with recombinant Skint-1 DV, and purified over a Pierce Sulfolink column, before concentration with Millipore Amicon-30 protein concentrator columns. One reactive clone was then purified using a ThermoScientific Nab Protein G spin column, concentrated with a Millipore Centricon Plus-70 centrifugal filter, and filtered using a Millipore 0.22-μm Ultrafree-MC centrifugal filter.

##### Western Blots

293 cells were transfected with pCAGGS constructs using polyethylenimine. Transfected cells were lysed in buffer (ice-cold 1% Nonidet P-40, 20 mm Tris-HCl, pH 7.8, 150 mm NaCl, 2 mm MgCl_2_, 1 mm EDTA, containing protease inhibitors) and the lysates were analyzed by Western blotting performed using standard protocols. The membranes were probed with either mouse anti-FLAG M2 or rat anti-Skint-1 antibodies.

##### Fetal Thymic Organ Culture

Fetal thymi were isolated from E14 wild type FVB mice. The thymic lobes were split and cultured in medium containing either rat IgG2a isotype control or rat anti-Skint-1 antibodies at 10 μg/ml. After 5 days, lobes from 1 litter were pooled and thymocyte maturation was assessed by flow cytometry using a BD LSRII flow cytometer and FlowJo software for analysis.

##### Reaggregate Thymic Organ Culture

Single cell suspensions were prepared from E14 to E15 fetal thymi by digestion with collagenase-dispase as described previously (19), and were spin-infected for 45 min with ×20 concentrated retrovirus from LinXE cells transfected with *Skint-1* constructs in MSCV-derived bicistronic vectors. Four thymic lobe equivalents were pelleted and the slurry filtered (Millipore) and then incubated at 37 °C with 10% CO_2_ to reaggregate. Finally, RTOC were disaggregated in collagenase-dispase, and stained with relevant antibodies, before flow cytometry using a FACS LSRII and analysis using FlowJo software.

##### Recombinant Skint-1 DV Expression and Refolding

A construct of *Skint-1* without the IgC and lacking the transmembrane spanning region (Ser^24^ to Thr^141^) was cloned into a pET23a expression vector (Novagen) and overexpressed in *Escherichia coli* BL21(DE3) strain (Novagen) in the presence of M9 minimal media supplemented with [^15^N]ammonium chloride and [^13^C]glucose. Expression was induced by the addition of 1 mm isopropyl β-d-1-thiogalactopyranoside for 16 h. The bacterial cell pellet was harvested by centrifugation using a Beckman Avanti J-26 XP centrifuge with a JLA 8.1 rotor at 5500 rpm for 20 min. Resuspension of the bacterial pellet was carried out using phosphate-buffered saline, lysis of cell solution was performed utilizing a Misonix sonicator 3000 (45 s, 1-s on 1-s off). The cell lysate was centrifuged at 13,500 rpm for 15 min to retrieve the overexpressed inclusion body protein. The pellet was washed three times in a Triton wash buffer (0.5% Triton X-100 (v/v), 200 mm NaCl, 10 mm EDTA, 0.01% sodium azide, and 50 mm Tris-HCl, pH 8.0) to remove any cellular debris. A final wash without Triton was followed by resuspension of the protein pellet into 8 m urea solubilization buffer. Skint-1 DV was renatured using dilution refolding in the presence of 5 m urea, 100 mm Tris, 0.4 m l-arginine-HCl, 2 mm EDTA, 0.5 mm oxidized glutathione, 5 mm reduced glutathione, and 0.1 mm PMSF, pH 8.3. Typically, 30 mg of Skint-1 DV protein was added to 1 liter of refolding buffer equilibrated to 4 °C over a period of 30 min. The refolding mixture was then incubated overnight at 4 °C, dialyzed against 100 mm urea overnight, and then dialyzed for a final time in 100 mm urea and 10 mm Tris, pH 8. The refolding mixture was concentrated down and purified by size exclusion chromatography using a Superdex-200 (GE Healthcare) column pre-equilibrated with 50 mm NaCl, 20 mm MES, pH 6.5. The Skint-1 DV protein displayed an elution profile that corresponded to a monomeric state in solution.

##### Surface Plasmon Resonance

*Skint-1 E. coli* protein expression constructs were mutated to substitute the CDR3 loop sequence of *Skint-1* with that of *Skint-2* (incorporating D127V and D129E mutations); separately, CDR3 loop residues Asp^127^ and Asp^129^, implicated as functionally important for DETC selection in RTOC experiments, were mutated to alanine in a single construct. Following DNA sequencing, constructs were expressed in *E. coli* as outlined above and enzymatically biotinylated via C-terminal biotinylation tags (20). Binding of Skint-1 mAb (10 μg/ml) to wild type and CDR3 mutant Skint-1 was then compared using surface plasmon resonance, carried out at 5 μl/min in HBS-EP buffer on a Biacore 3000 essentially as previously described (21). Briefly, Skint-1 mAb was injected over SAcaps chips and responses were observed over surfaces to which biotinylated wild-type Skint-1 and Skint-1 bearing CDR3 loop mutations had been immobilized, and a control surface.

##### Nuclear Magnetic Resonance Spectroscopy

NMR experiments were performed at 303 K on Varian Inova 600 and 800 MHz NMR spectrometers equipped with triple resonance cryogenic probes and *z* axis pulse field gradients. The concentration of Skint-1 DV was 1.4 mm in 20 mm MES, pH 6.5, and 50 mm NaCl. Backbone assignments were made from BEST versions of ^15^N-HSQC, CBCA(CO)NH, HNCACB, HNCA, HN(CO)CA, HNCO, HN(CA)CO, H(C)CH TOCSY, (H)CCH TOCSY, ^15^N-edited NOESY-HSQC (τ_mix_ = 100 ms), and ^13^C-edited NOESY-HSQC experiments (τ_mix_ = 100 ms) (22, 23). Asn and Gln side chain ^1^H and ^15^N resonances were assigned using three-dimensional ^15^N-edited NOESY-HSQC and three-dimensional CBCA(CO)NH spectra. Spectra were processed using NMRPipe (24) and analyzed using SPARKY (T.D Goddard and D.G. Kneller, University of California, San Francisco).

##### Structure Calculation

Proton distance constraints for the structure calculation were obtained through the analysis of three-dimensional ^15^N-edited (τ_mix_ = 100 ms) and ^13^C-edited (τ_mix_ = 100 ms) NOESY-HSQC spectra. The cross-peaks were manually picked and the peak volumes estimated using SPARKY (25). Backbone dihedral angle restraints (ϕ and ψ) were obtained using the TALOS method (26). The three-dimensional structure was determined using the CANDID/CYANA package with the automated NOE cross-peak assignment and structure calculation with torsion angle dynamics implemented (27). The CYANA protocol consisted of seven iterative cycles of NOE assignment and structure calculation. The first cycle starts from 100 random conformers with 10,000 torsion angle dynamic steps performed per conformer in each cycle with the results being used as input in the following cycle. The seven cycles were followed by a final structure calculation where the 20 conformers with the lowest CYANA target function were chosen as representative. Aria1.2 was used to perform the final water minimization (28). A total of 828 NOEs, 50 hydrogen bonds, and 180 dihedral angle restraints were used in the final calculation. Structures were analyzed using PROCHECK-NMR (29). The structural statistics for Skint-1 DV are listed in Table 1. Root mean square deviation (r.m.s. deviation) values from the average structure were calculated using the MOLMOL program (30). The structural coordinates for Skint-1 DV have been deposited in the RCSB Protein Data Bank under accession code 2N4I.

## Results

### 

#### 

##### An Antibody to Skint-1 Prevents Vγ5Vδ1 T Cell Maturation

**FIGURE 1.**
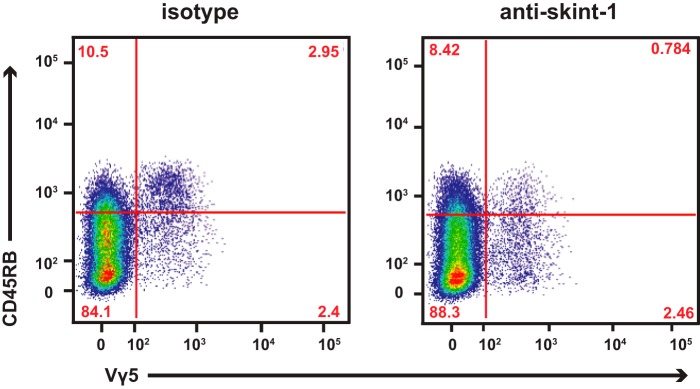
**An antibody specific for the Skint-1 ectodomain blocks DETC progenitor maturation in FTOC.** Addition of anti-Skint-1 DV antibody in FTOC experiments (*right panel*) substantially decreased the efficiency of DETC selection compared with addition of isotype control (*left panel*).

*Skint-1* expression in thymic epithelial cells is essential for DETC selection (15, 16). Although *Skint-1* mRNA is readily detectable in thymic medullary epithelial cells (17, 31), albeit at different levels in different strains of mice, it has not proved possible to reliably detect Skint-1 protein at the surface of cells, formally casting doubt on whether it mediates its effects on DETC progenitors via cell surface interactions. To investigate this issue, we generated a monoclonal antibody that proved to be specific to the Skint-1 ectodomain (Fig. 1, supplemental Fig. S2). In early fetal thymus development, the acquisition of CD45RB is a maturation marker for TCRγδ^+^ thymocytes, the majority of which are Vγ5Vδ1 DETC progenitors (16). Thus, >50% of TCRγδ^+^ thymocytes in fetal thymic organ culture (FTOC) show Skint-1-dependent maturation, which was also the case in FTOC supplemented with isotype control antibody (Fig. 1, *left panel*). However, in FTOC supplemented with anti-Skint-1-DV mAb, there was a marked inhibition of maturation, and likewise the number of TCRγδ^+^ events was reduced, consistent with anti-Skint-1 inhibiting *Skint1-*dependent maturation and selective expansion of DETC progenitors (Fig. 1, *right panel*). This impairment of maturation, as reflected in a reduced ratio of CD45RB^+^:CD45RB^lo^ DETC progenitors was invariably observed over three additional, independent experiments (supplemental Fig. S3). Collectively these data provide evidence that Skint-1 function requires its surface interaction with a counter-receptor, most likely on DETC progenitors. Consistent with this, Skint-1 deficiency can be rescued with DETC TCR agonists (16).

##### Residues on the Membrane-Distal Variable Domain of Skint-1 Are Critical for DETC Selection in RTOC

**FIGURE 2.**
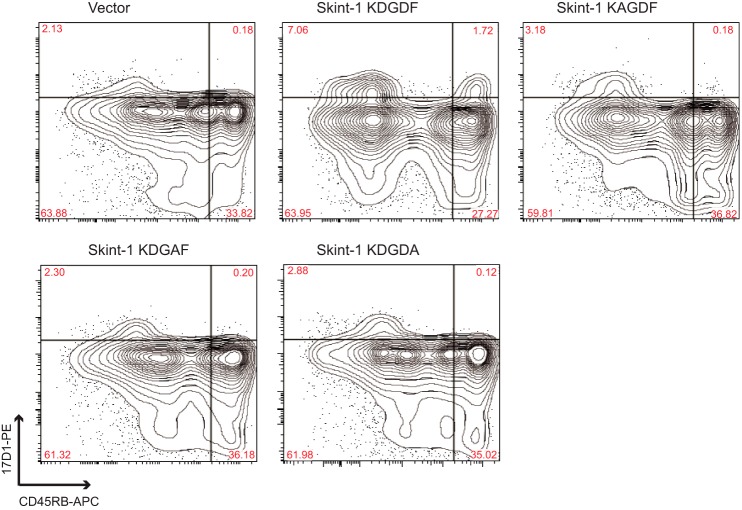
**Residues in the Skint-1 CDR3 loop are critical for DETC selection.** E15 Tac fetal thymi were disaggregated via trypsin and infected with concentrated retrovirus encoding *Skint-1* or *Skint-1*-CDR3 mutants by spinoculation. Infected cells were then reaggregated and cultured for 12 days prior to disaggregation, stained with CD45RB-APC and 17D1-PE antibodies, and analyzed by flow cytometry.

To understand the potential role of IgV domain features in DETC selection, we studied Skint-1/2 sequence alignments, which showed the Skint-1 DV CDR3-like loop contained several residues that were not conserved in Skint-2, and therefore could be important for Skint-1 biological activity. Three CDR3 amino acids, Asp^127^, Asp^129^, and Phe^130^, when mutated individually to alanine, each failed to support development of mature, CD45RB^+^17D1^+^ DETC (Fig. 2), indicating multiple residues in the CDR3-like loop region of Skint-1 are critical for DETC selection.

To address whether Skint-1 mAb blocked DETC selection by binding to this putative binding site, we mutated the CDR3 loop of Skint-1 to that of Skint-2 (incorporating D127V and D129E), and separately incorporated two alanine substitutions in residues Asp^127^ and Asp^129^, which individually abolished Skint-1-mediated DETC selection in RTOC. In each case, binding of the anti-Skint-1 mAb we raised was preserved. These data suggest the Skint-1 mAb generated abrogates Skint-1-mediated DETC selection most likely by steric effects, and does not appear to abrogate selection by binding to the putative ligand binding site we identified (supplemental Fig. S4).

##### Solution Structure of Skint-1 DV

**FIGURE 3.**
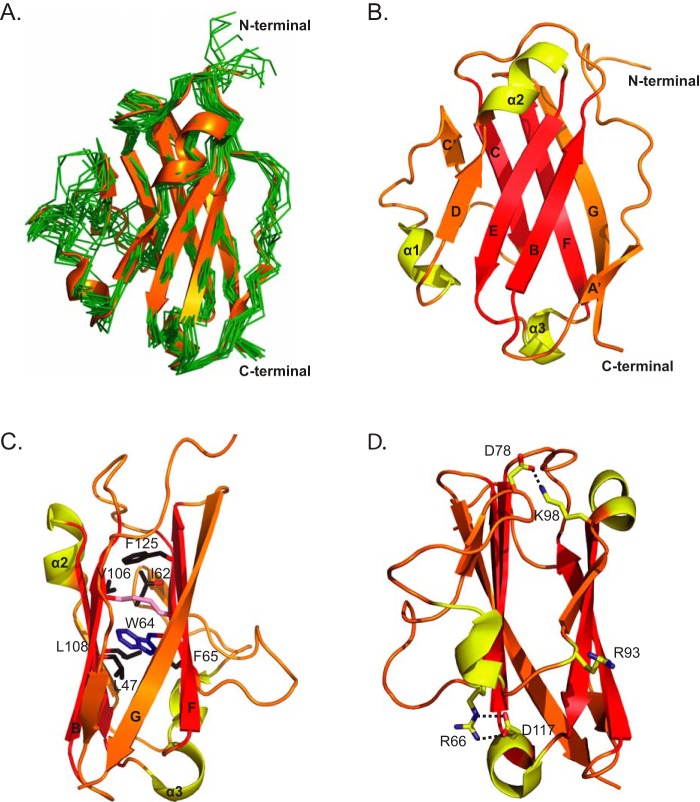
**Overall structure of Skint-1 DV.** *A*, superposition of 20 solution structures of the membrane-distal Skint-1 DV domain with each state displayed as Cα traces (*green*). The lowest energy structure is shown as a ribbon (*orange*). *B,* ribbon representation of the lowest energy NMR structure of Skint-1 DV, with strands colored *red* involved in stabilizing the IgV core region. Helices are shown in *yellow*. The β-strands are labeled according to the IgV-type fold (40). *C,* the core packing is mediated by β sheet residues (*black sticks*) from the B, E, F, and C strands (*red*) and is stabilized by an intra-chain disulfide bond between Cys^49^ and Cys^123^ (*pink*), and an invariant tryptophan residue (*blue*). *D*, polar residues involved in maintaining the IgV-fold are highlighted. *Black dashed lines* indicate hydrogen bonding interactions.

Given its unique capacity to promote DETC progenitor maturation, we solved the structure of Skint-1 DV by nuclear magnetic resonance spectroscopy. The final ensemble of 20 structures converged with a mean backbone r.m.s. deviation of 0.8 Å for the structured regions (Fig. 3*A*). The Skint-1 DV adopts a compact β sandwich domain comprising two anti-parallel sheets (the front sheet consisting of strands A′, B, D, E, and back sheet encompassing strands C, C′, F, G), with three helices positioned between the C′-D, D-E, and E-F strand pairings, and N and C termini at opposite ends of the molecule (Fig. 3*B*). Structural features that stabilize the overall fold of the domain include a large hydrophobic core formed by non-polar residues from strands B, C, and F (Fig. 3*C*), and also the packing of the invariant Trp^64^ from strand C against the intrachain disulfide bond (mediated between Cys^49^ and Cys^123^), which represents a hallmark of the Ig fold. Finally two salt bridges involving Arg^66^/Asp^117^ and Asp^78^/Lys^98^ help stabilize the domain (Fig. 3*D*); interestingly, these appear to be broadly conserved across the Skint family, and likely represent a distinctive feature of Skint receptors in that equivalent salt bridges are not found in other IgV domains analyzed structurally to date. Considerable disorder is observed throughout the loop regions and particularly the α3 helix and the N-terminal region as evidenced by the low S^2^ values on analysis of the backbone order parameters as calculated from the TALOS package (supplemental Fig. S5). The elevated dynamics here are consistent with the low number of Nuclear Overhauser Effect (NOE) signals observed (Table 1), suggesting the molecule is quite flexible in solution.

**TABLE 1:** **NMR statistics for the Skint-1 DV structure ensemble**

	Skint-1 DV*^a^*
**Completeness of resonance assignments*^b^***	
Aromatic completeness	60.71
Backbone completeness	88.22
Side chain completeness	79.29
Unambiguous CH2 completeness	100
Unambiguous CH3 completeness	100
Unambiguous side chain NH**_2_** completeness	100

**Conformationally restricting restraints*^c^***	
Distance restraints	
Total NOEs	828
Intra residue ( ***i = j***)	92
Sequential (| ***i–j*** | = 1)	177
Medium range (1 < | ***i - j*** | < 5)	136
Long range (| ***i–j*** | **≥** 5)	423
Dihedral angle restraints	180
Hydrogen bond restraints	50
No. of restraints per residue	9.4
No. of long range restraints per residue	4.1

**Residual restraint violations*^c^***	
Average No. of distance violations per structure	
0.2 Å-0.5 Å	0.95
> 0.5 Å	0
Average No. of dihedral angle violations per structure	
> **5^o^**	0

**Model quality*^c^***	
R.m.s. deviation backbone atoms (Å)*^d^*	0.8
R.m.s. deviation heavy atoms (Å)*^d^*	1.3
R.m.s. deviation bond lengths (Å)	0.005
R.m.s. deviation bond angles (**^o^)**	0.6

**Procheck Ramachandran statistics*^c,d^***	
Most favored regions (%)	88.1
Additionally allowed regions (%)	10.9
Generously allowed regions (%)	0.8
Disallowed regions (%)	0.2

**Global quality scores (raw/Z score)*^c^***	
Verify 3D	0.35/−1.77
Prosall	0.12/−2.19
Procheck (φ-ψ)*^d^*	−0.71/−2.48
Procheck (all)*^d^*	−1.15/−6.80
Molprobity clash score	34.19/−4.34

**Model contents**	
Ordered residue ranges*^d^*	7–115
Total number of residues	119
BMRB accession number	17833
PDB code	2N4I

*^a^* Structural statistics computed for the ensemble of 20 deposited structures.

*^b^* Computed using AVS software (41) from the expected number of resonances, excluding highly exchangeable protons (N-terminal, Lys, amino and Arg guanido groups, hydroxyls of Ser, Thr, and Tyr), carboxyls of Asp and Glu and non-protonated aromatic carbons.

*^c^* Calculated using PSVS version 1.5 (42). Average distance violations were calculated using the sum over r^−6^.

*^d^* Based on ordered residue ranges (S(ϕ) + S(ψ) > 1.8).

##### Comparisons of Skint-1 with Structural Homologues

**FIGURE 4.**
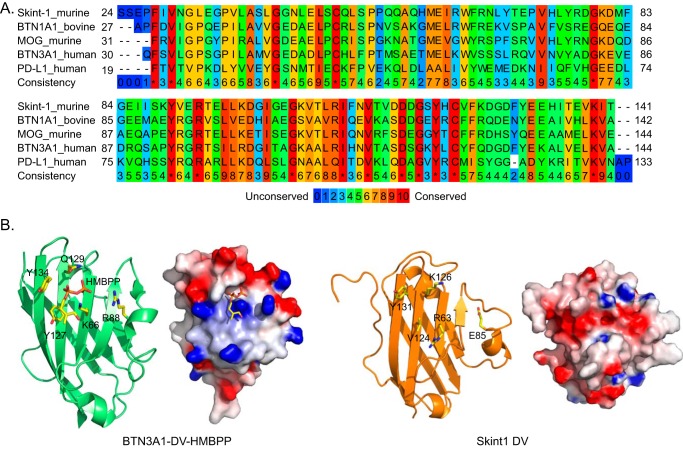
**Comparison of Skint-1 DV with structural homologues.** *A,* multiple sequence alignment of the IgV domains of murine Skint-1 with DALI-derived structural homologues. Sequences were downloaded from the Uniprot database (accession entry; BTN1A1_bovine (P18892), BTN3A1_human (O00481), MOG_murine (Q61885), Skint-1_murine (A7TZE6), and PD-L1_human (Q9ZQ7)) and aligned with Praline. The color scheme of the alignment is for amino acid conservation. *B,* comparison of the surface-exposed grooves between BTN3A1 and Skint-1. *Left panels*, ribbon diagram for the BTN3A1-HMBPP complex (PDB code 4K55, *lime*). *Right panel*, ribbon representation of the equivalent region of Skint-1 (*orange*). The electrostatic potential for each structure is also highlighted. The scale ranges from −7 (negative potential, red) to +7 (positive potential, *blue*) in units of kT/e.

Structural comparisons using DALI (32) identified a number of Ig V domain structural homologues of Skint-1 DV (Fig. 4*A*), including extracellular domains of bovine butyrophilin subfamily 1 member A1 (BTN1A1; PDB code 4HH8; Z score 13.2, r.m.s. deviation 2.1 Å), murine myelin oligodendrocyte (MOG; PDB code 1PY9; Z score 12.8, r.m.s. deviation 2.3 Å (33)), human butyrophilin subfamily 3 member A3 (BTN3A3; PDB code 4F8T; Z score 12.4; r.m.s. deviation 2.5 Å (34)), human butyrophilin subfamily 3 member A1 (BTN3A1; PDB code 4F9P; Z score 12.3; r.m.s. deviation 2.5 Å (34)), and human programmed death-ligand 1 (PD-L1; PDB code 3FN3; Z score 12.2; r.m.s. deviation 2.4 Å (35)). These ranged from 21 to 45% identity to the Skint-1 DV sequence (Fig. 4*A*).

First, we compared the structure of Skint-1 DV with its closest structural homologue, BTN3A1, which plays a key role in phosphoantigen-mediated activation of human peripheral blood γδ T cells (36). Vavassori and colleagues (37) presented crystal structures of the IgV domain of BTN3A1, identifying a shallow groove on the protein surface, which they reported to accommodate a single phosphoantigen, P-Ag (Fig. 4*B, left*). Although direct engagement of P-Ag has been disputed by others (38), we note that the equivalent region of Skint-1 also contains a shallow groove, leaving open the possibility of accommodating the low molecular mass cargo (Fig. 4*B, right*). Of note, however, the Skint1 groove lies close to a significant strip of electronegative charge (perpendicular to the long axis of the G, F, and C strands), which would be energetically unfavorable for binding negatively charged polar moieties (Fig. 4*B, right*).

Comparison revealed topological differences between Skint-1 and its homologues. First, the hydrogen-deuterium exchange analysis of Skint-1 demonstrates that although the N-terminal region up to the strand A′ is structurally defined via NOE restraints, it is weakly associated with the structure, as no protection of exchangeable amides was notable. Consequently, this segment of Skint-1 is devoid of regular secondary structure, adopting a random coil conformation rather than the short strand A that packs against strand G in all homologous structures (supplemental Fig. S6). Consistent with this, analysis of the disorder across the domain using the TALOS software highlighted this A strand region as particularly mobile (supplemental Fig. S5). Second, in Skint-1 DV the short strand termed C″ is replaced by an extremely flexible loop; also whereas, MOG and PD-L1 possess two 3^10^ helices immediately following this C″ strand, Skint-1 and the BTN family members retain a single α helical segment (supplemental Fig. S6). Comparisons with MOG and PD-L1 also revealed that the mode of dimerization they adopt would be structurally implausible for Skint-1 (supplemental Fig. S7). Thus, whereas the MOG dimer interface is dominated by contacts between residues projecting from the C-C′ loop of opposing monomers (supplemental Fig. S7*A*, *left*), the C-C′ loop for Skint-1 is more extended and orientated at a perpendicular angle that protrudes from the β sheet. The ensuing steric clashes that would occur with the corresponding C-C′ loop of the opposing Skint-1 monomer would preclude this mode of dimerization (supplemental Fig. S7*A*, *right*). In both its native state in solution and in its crystalline state, the ectodomain of PD-L1 forms homodimers (35) via an interface that is dominated by hydrogen-bonding interactions mediated by strand A (Thr^22^), strand B (Glu^39^ and Lys^41^), B-C loop (Val^44^), and α4 (Gln^91^) residues (supplemental Fig. S7*B*, *left*). Of note, the majority of residues that stabilize this PD-L1 dimer interface are non-conservatively substituted in the equivalent regions of Skint-1 (supplemental Fig. S7*B*, *right*), strongly suggesting that the molecular basis for PD-L1 dimerization cannot pertain to Skint-1.

##### Identification of a Putative Interaction Surface on Skint-1

**FIGURE 5.**
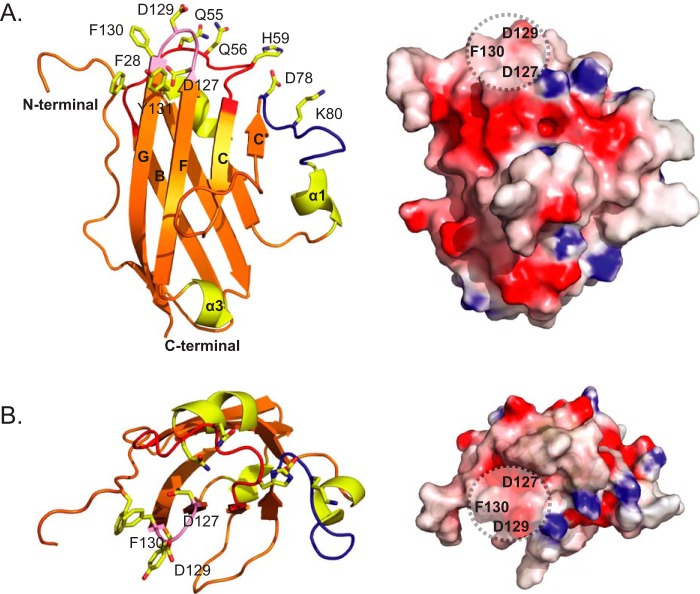
**Putative receptor binding surface for Skint-1 DV.** *A, left panel*, schematic representation of the membrane-distal region of Skint-1 (*orange*). The random coil regions that connect the B-C (*red*), C′-α1 (*blue*), and F-G (*pink*) strands have been termed CDR-1/2/3-like loops, respectively. CDR-like loop residues that are extremely prominent are highlighted in stick format. *Right panel*, electrostatic potential for Skint-1 orientated as *left panel*. The three CDR-like loops combine to form a charged and undulating molecular surface. The scale ranges from −7 (negative potential, *red*) to +7 (positive potential, *blue*) in units of kT/e. *B, left panel*, alternative top view of the membrane-distal region of Skint-1. *Right panel*, electrostatic potential for Skint-1 orientated as *left panel*. The putative counter-receptor binding patch is highlighted (*black dashed circle*).

The membrane-distal end of Skint-1 is characterized by three loops that connect the B-C (Ser^52^-His^59^), C′-α1 (Gly^79^-Glu^85^), and F-G (Asp^127^-Phe^130^) strands, equivalent to the complementarity determining regions (CDR)-1/-2/-3 of IgV domains present in TCRs and antibodies (Fig. 5*A*, *left*), respectively. In Skint-1 DV, these loops collectively present an undulating and charged molecular surface potentially available for ligand binding (Fig. 5*A*, *right*). The CDR2-like loop of Skint-1, which contains both hydrophobic and hydrophilic residues, is more extended relative to the corresponding region of other IgV family members because of the absence of the C″ region. The CDR3-like loop is located in a highly prominent solvent-exposed position of the IgV domain, with two solvent-exposed aromatic hydrophobic residues (Phe^130^ and Tyr^131^) located on and around the CDR3-like loop (Fig. 5*B*). Based on the recognized propensity of exposed hydrophobic residues to be in involved in protein-protein recognition interfaces, and implication of the CDR3-like loop in Skint-1-mediated functions, this region of Skint-1 is a candidate for contributing to the ligand recognition surface (Fig. 5*B*).

##### Comparison of the Skint-1 DV with Other Skint Gene Family Members

**FIGURE 6.**
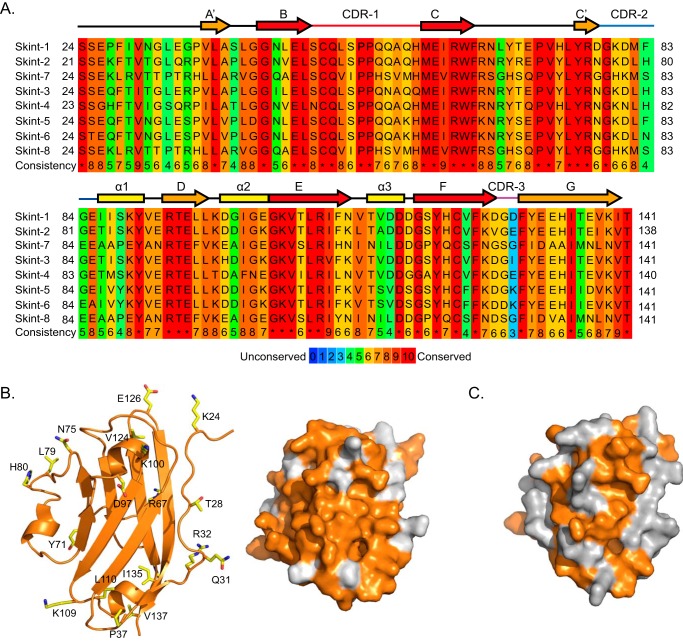
**Sequence and structural comparison of the IgV domains of Skint family members 1–8.** *A,* sequences of IgV domains were downloaded from the Uniprot database (accession numbers; Skint-1 (A7TZE6), Skint-2 (A7XUX6), Skint-3 (A7TZF0), Skint-4 (A7TZF3), Skint-5 (A7XUY5), Skint-6 (A7XUZ6), Skint-7 (A7XV04), and Skint-8 (A7XV07)) and aligned with Praline. The color scheme of the alignment is for amino acid conservation. Skint-1 DV structure based secondary structural elements are color coded as described in the legend to Fig. 3*B* (β sheets are shown as *arrows* and helices are highlighted as *rectangles*). *B*, *left panel*, schematic representation of Skint-1 IgV (*orange*) with Skint-2 residues mapped (highlighted in stick format). The majority of the substitutions are solvent exposed. *Right panel*, molecular surface representation of Skint-1 (*orange*) highlighting surface amino acid differences with Skint-2 (*gray*). *C*, molecular surface representation of Skint-1 (*orange*) highlighting surface amino acid differences with Skint-7 (*gray*).

The Skint family can be divided into three subfamilies based on sequence identity to Skint-1: those bearing high sequence similarity (75–81% identity, Skint-2 to -6); moderate similarity (46–47% identity, Skint-7 and -8), and low similarity (Skint-9 to -11; 8–10% identity; alignment data not shown) (Fig. 6*A*). Initial analyses mapping highly conserved segments onto the Skint-1 structure revealed that these corresponded to core secondary structural elements that help stabilize the classical IgV domain, suggesting that many Skint family members may adopt a similar core-fold (Fig. 6*A*). In contrast, the least conserved regions were mainly restricted to the loops that interconnect the β strands, particularly the CDR3-like loop, which is implicated in the biological activity of Skint-1.

**FIGURE 7.**
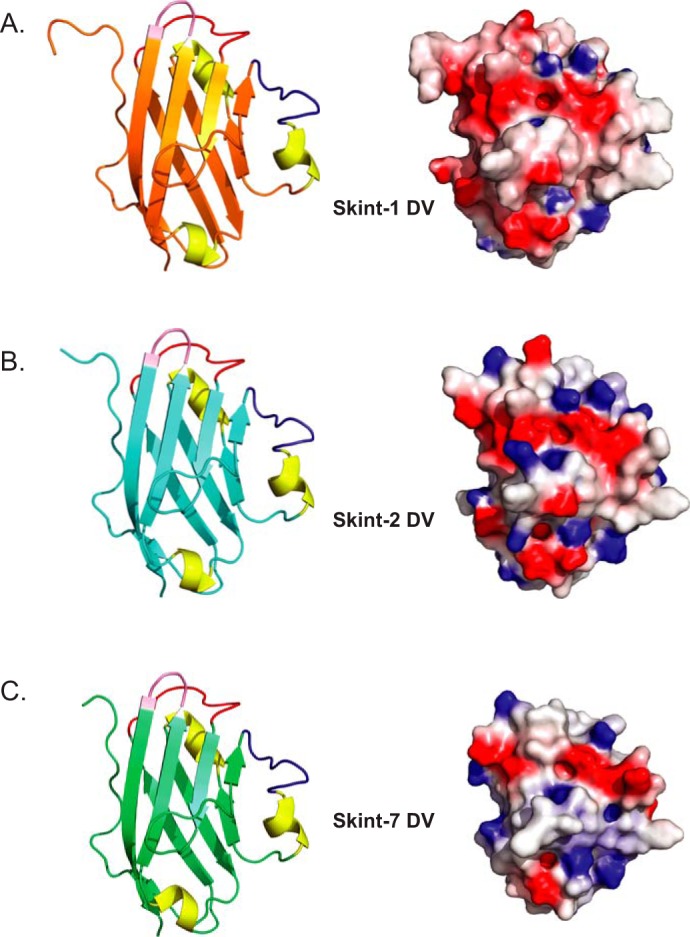
**Structural comparison of Skint-1 with Skint-2 and Skint-7.** *A,* overall structure of Skint-1 DV. *B,* model of Skint-2 DV derived from Phyre. *C,* model of Skint-7 DV derived from Phyre. For each domain the CDR-like loops are highlighted (CDR-1 (*red*), CDR-2 (*blue*), and CDR-3 (*pink*)). *Right panels* highlight electrostatic potential for each structure and are orientated as the *left panel*. The scale ranges from −7 (negative potential, *red*) to +7 (positive potential, *blue*) in units of kT/e.

We then explored how sequence conservation mapped onto the Skint-1 structure (Fig. 6, *B* and *C*). Notably, sequence comparisons between Skint-1 and Skint-2 variable domains highlighted 23 amino acid substitutions. Of note, the majority of these (78%), when mapped onto the Skint-1 structure, are predicted to be solvent exposed (Fig. 6*B*). This observation extended across the family, with 78–88% of substitutions relative to Skint-1 occupying solvent-exposed positions (Fig. 6*C*). These data show that the Skint-1 DV core tertiary structure is likely to be highly conserved across the entire Skint family, providing a sound basis for modeling the equivalent domains of other family members. On this basis, we used the PHYRE modeling server to generate molecular models of Skint-2 and Skint-7 (Fig. 7, *A–C*), which are conserved alongside Skint-1 in other rodents, and found they each had highly distinct surface electrostatic profiles, with both Skint-2 and Skint-7 exhibiting more electropositive patches compared with Skint-1 (Fig. 7, *B* and *C*). This would be consistent with unique functions of these molecules.

##### Comparison of the CDR-like Loops of Skint-1 with Skint-2 and Skint-7

**FIGURE 8.**
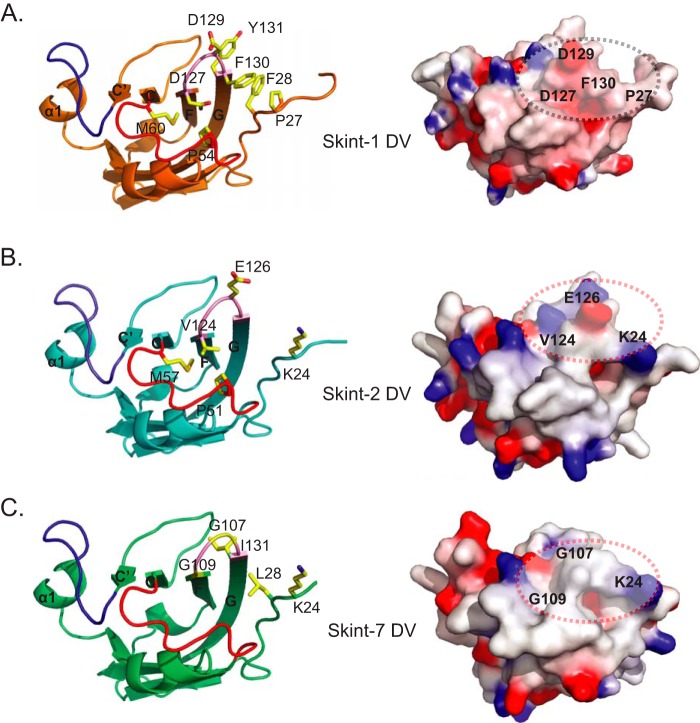
**Structural comparison of the membrane-distal regions of Skint-1 with Skint-2 and Skint-7.** *A,* schematic representation of the membrane-distal region of Skint-1 (*orange*). Residues that are predicted to be critical for DETC selection are highlighted. *B,* schematic representation of the membrane-distal region of the Phyre-derived Skint-2 model (*cyan*). Residues that are predicted to perturb binding to the DETC selecting ligand are highlighted. *C,* schematic representation of the membrane-distal region of Phyre-derived Skint-7 model (*lime green*). Residues that may prevent binding to DETC selection are highlighted. For all structures the CDR-1/2/3-like loops are highlighted in *red*, *blue*, and *pink*, respectively. *Right panels* highlight distinct electrostatic potential for each structure and are orientated as the *left panel*. The scale ranges from −7 (negative potential, *red*) to +7 (positive potential, *blue*) in units of kT/e. The *black dashed circle* represents the Skint-1 putative receptor binding surface and the *red dashed circles* highlight the equivalent regions of Skint-2 and Skint-7.

Our RTOC experiments indicated that Skint-1 constructs featuring individual alanine mutants of Asp^127^ and Asp^129^ in the CDR3-like loop failed to rescue development of DETC, thereby confirming the functional significance of each negatively charged moiety. Availability of the Skint-1 DV structure enabled us to assign the molecular role of each of these critical residues in the domain, and to compare the molecular features of Skint-1 in this region to some of its key intra-family homologues. Most strikingly, the Skint-1 side chain of Asp^129^ is prominently exposed on the membrane-distal tip of the domain, and makes no contact with other amino acid side chains, thereby suggesting that it interacts with other molecules (Fig. 8*A*). In contrast, the negatively charged Asp^127^ residue in Skint-1 is partially buried in a hydrophobic environment contributed by the aliphatic groups of Pro^54^, Met^60^ (from the CDR2-like loop), and the aromatic ring of Phe^130^ (CDR3-like loop) (Fig. 8*A*). In Skint-2, Asp^127^ is replaced by Val^124^, a non-conservative alteration that could allow Val^124^ to generate extensive hydrophobic interactions with nearby non-polar side chains of Pro^51^, Met^57^, and Phe^127^, and that may therefore affect the conformation in this critical region (Fig. 8*B*). Moreover, although Skint-2 preserves the charge of Asp^129^ (substituted by Glu^127^ in Skint-2), the longer Glu^127^ side chain might prevent it engaging a putative counter-receptor (Fig. 8*B*). By comparison to Skint-1, Skint-7 contains two non-conservative substitutions for the negatively charged CDR3-like loop residues at positions 127 and 129 (to glycine in each case), suggesting that Skint-7 presents a highly flexible and relatively featureless CDR3-like loop relative to Skint-1 (Fig. 8*C*).

In addition to these differences, Skint-1 also possesses a proline residue (Pro^27^), which is located near the CDR3-like loop and two solvent-exposed hydrophobes, and is not present in any other Skint family member (Fig. 8*A*). Importantly, this proline appears to reverse the direction of the Skint-1 main chain away from the putative ligand binding site and the CDR3-like loop. The corresponding position in Skint-2 and -7 is occupied by a long and positively charged side chain (Lys^24^ in Skint-2 and Lys^27^ in Skint-7) (Fig. 8, *B* and *C*). Collectively, these observations provide molecular evidence that Skint-1 possesses highly distinctive features in the membrane-distal region of its variable domain around the CDR3-like loop, which are not preserved in other family members and may help to explain the non-redundant role of Skint-1 in facilitating the positive selection and development of DETC.

## Discussion

*Skint-1* is the first identified putative selecting element for a major γδ T cell compartment in rodents. Moreover, although all hominoid species appear to have inactivating mutations in the *Skint-1-*like gene, recent analyses suggest Old World primates do have apparently functional *Skint-1* genes, and may possess dendritic epidermal T cell populations (39). As such, it promises to be of general relevance to other intraepithelial compartments and/or other non-MHC restricted T cells (15, 16), in which regard, its biology may inform that of its relative, BTN3A1, which is essential for human peripheral blood γδ T cell recognition of so-called P-Ags (36). Thus, it is important to characterize the molecular mechanisms whereby Skint-1 exerts its non-redundant effects.

First, our finding that an antibody to Skint-1 impairs DETC selection in fetal thymic organ culture argues that cell surface expression is indeed crucial for Skint-1 function. Although impairment was not complete, this may reflect both the steric challenge of blocking an avid interaction between two closely aligned cells, and the possibility that the antibody binds in a manner that inhibits but does not fully block counter-receptor engagement. The latter possibility is consistent with our finding that mutations in the CDR3 loop of Skint-1 that disrupt DETC selection do not affect Skint-1 mAb binding, suggesting it most likely inhibits DETC selection via a steric blocking mechanism. Given that the antibody is specific for Skint-1 DV, the findings are consistent with studies on Skint-1/2 chimeras that reported the functional importance of the Skint-1 V-domain in DETC selection (17). This perspective is further supported by the identification in this study of two functionally important residues in the CDR3-like loop at the membrane-distal tip of the V-domain, and by our domain alignments that highlight the Skint-1 CDR3-like loop as being distinct compared with other family members.

Our first analysis of a Skint family structure provided insight into the molecular basis of the effects of Skint-1. In addition to highlighting retention of many classical features of the IgV domain-fold, the structure highlighted several distinct features that contrast Skint-1 with its structural homologs. First, Skint-1 DV is unlikely to mediate homodimer formation in a manner similar to either MOG or PD-L1, due to steric clashes and molecular alterations at the potential Skint-1 dimer interface, respectively. Consistent with this, Skint-1 DV exists as a monomeric form in solution, as highlighted by size exclusion profiles during purification. Nevertheless, Skint-1 may dimerize through other mechanisms, for example, via its constant-like domain, as observed for members of the BTN family (34). Second, comparison of surface electrostatic patterns of the IgV modules of Skint-1 and MOG highlighted several regions encompassing distinctive electronegative patches. Finally, and most relevant to our RTOC results, whereas in MOG the CDR-like loop regions combine to form a flat and uncharged surface, in Skint-1 they combine to form an undulating and charged surface, with the CDR3-like loop occupying a particularly prominent position. Furthermore, located on or around the CDR3-like loop are several solvent-exposed aromatic hydrophobic residues (including Phe^130^ and Tyr^131^), a common feature of protein-protein docking sites. These considerations distinguish this membrane-distal region as a candidate receptor interaction surface.

Determination of the Skint-1 DV structure enabled us to generate structural models of other Skint family members, using standard structure-based modeling algorithms. In particular, we generated structural models of Skint-2 and Skint-7 using the solution structure of Skint-1 DV as a template. These models suggest a largely conserved tertiary architecture for the Skint-1/2/7 DV domains. However, the majority of the non-conserved residues between these molecules were restricted to the surface of the IgV molecule. Consistent with this, Skint-2 and Skint-7 exhibited a series of distinctive electropositive patches that were absent in Skint-1. In sum, these findings argue that the non-redundant role in DETC selection are underpinned by distinct electrostatic and chemical features of the Skint-1 DV surface, rather than by gross conformational differences relative to Skint-2 and Skint-7.

The availability of the Skint-1 DV structure, along with models of other Skint family members, also provided a basis to examine potential molecular roles of residues in the CDR3-like loop of Skint-1, including those that were implicated in Skint-1-mediated DETC selection by RTOC experiments. Notably, the CDR3-like regions of Skint-1 and Skint-2 are highly conserved with the exception of two residues at positions 127 and 129 (aspartic acid at both positions in Skint-1; a valine and glutamic acid, respectively, in Skint-2). Individual alanine mutations in Skint-1 at each of these residues abolished selection in RTOC. Most strikingly, Asp^129^, located at the central region of the CDR3-like loop, is entirely solvent exposed and makes no contact to other residues. In addition, Phe^130^, conserved across the Skint-1 family, is largely solvent exposed, and abolishes DETC selection when substituted to alanine. In contrast, Asp^127^ is partially buried in the Skint-1 structure and interacts with neighboring hydrophobic residues: substitution to alanine at this position (or to the equivalent valine found in Skint-2) could conceivably have effects on overall conformation in this region. However, the fact that alanine substitutions at entirely exposed residues in the membrane-distal CDR3 loop, specifically Asp^129^ and Phe^130^, abolish DETC selection is strongly suggestive of Skint-1 interaction with a counter-receptor, although we cannot exclude that these affect Skint-1 association in *cis* with other components on Skint-1-expressing cells. Clearly, identification of Skint-1 ligand(s) is required to understand these issues, and to fully appreciate the molecular role of the CDR3 loop that so strongly affects Skint-1-mediated DETC selection.

An obvious possibility is that this putative interaction surface on Skint-1 interacts with the Vγ5Vδ1 TCR expressed on DETC, consistent with our previous suggestion that Skint-1 is likely to bind a target structure on DETC (17). However, in several experiments we have failed to observe direct binding between recombinant Vγ5Vδ1 TCRs and Skint-1 DV (data not shown). Current reports suggest an analogous situation for human BTN3A1 and TCRVγ9Vδ2. Among possible explanations are first, that there are other, undefined molecular components that together with Skint-1 form an active ligand complex for the TCR; second, that any interaction is of extremely low affinity.

In sum, our results indicate that the non-redundant role of Skint-1 in DETC selection depends critically on cell surface expression on thymic epithelial cells, and specifically on solvent-exposed residues on the membrane-distal tip of Skint-1 DV, highly suggestive of an interaction with a counter-receptor on target cells.

## Author Contributions

M. S., T. J. K., and R. H. designed the study and carried out experiments. F. M., M. J. W., C. R. W., and M. O. analyzed data and wrote the manuscript. B. E. W. and A. H. designed the study, analyzed data, and wrote the manuscript.

## Supplementary Material

Supplemental Data

## Footnotes

5 The abbreviations used are:

DETC: dendritic epidermal T cells
TCR: T cell receptor
NKT: natural killer T
IgV: immunoglobulin variable
IgC: immunoglobulin constant
r.m.s.: root mean square
FTOC: fetal thymic organ culture
CDR: complementarity determining regions
MOG: myelin oligodendrocyte
RTOC: reaggregate thymic organ culture
Skint-1 DV: variable domain of Skint-1.
