# Supplementary material for: Characterization of a Putative Receptor Binding Surface on Skint-1, a Critical Determinant of Dendritic Epidermal T Cell Selection
Source: J Biol Chem. 2016 Feb 25;291(17):9310–21. doi: 10.1074/jbc.M116.722066 (PMC4861494; doi:10.1074/jbc.M116.722066)
Supplement: Supplemental Data [file supp_291_17_9310__index.html]

Characterisation of a putative receptor binding surface on Skint-1, a critical determinant of dendritic epidermal T cell selection — Characterization of a Putative Receptor Binding Surface on Skint-1, a Critical Determinant of Dendritic Epidermal T Cell Selection — Structure-Function Studies of Skint-1 — Supplemental Data 

# Characterization of a Putative Receptor Binding Surface on Skint-1, a Critical Determinant of Dendritic Epidermal T Cell Selection

## Supplemental Data

- 4 (.pdf, 2.2 MB) - Supplementary Figures 1-7
