# Supplementary material for: Characterization of a Putative Receptor Binding Surface on Skint-1, a Critical Determinant of Dendritic Epidermal T Cell Selection
Source: J Biol Chem. 2016 Feb 25;291(17):9310–21. doi: 10.1074/jbc.M116.722066 (PMC4861494; doi:10.1074/jbc.M116.722066)
Supplement: Supplemental Data [file 10.1074_M116.722066_jbc.M116.722066-1.pdf]

Supplementary Figure 1. Multiple features of Skint-1 are important for its role in DETC selection. Schematic of Skint-1, indicating the signal peptide (purple diamond), extracellular immunoglobulin Variable (orange) and Constant-like (green) domains and membrane spanning region (red). The putative location of the E324X mutation that abolishes DETC selection in FVBtac is indicated (red arrow). Domain deletion experiments established that multiple regions of Skint-1 protein are important for DETC selection.

Supplementary Figure 2. Validation of the anti-Skint-1 mAb
Western blot analysis of 293 cells transfected with N-terminal FLAG-tagged Skint-1, Skint-2 or Skint-7 constructs. All three proteins are detected by anti-FLAG mAb (left panel), but the Skint-1 mAb (right panel) only detects 293 cells expressing Skint-1. 293 tranfection and Western blotting was carried out essentially as described (1).

Supplementary Figure 3. Effects of anti-Skint-1 mAb on DETC maturation
Three independent foetal thymic organ culture experiments carried out in the presence and absence of the anti-Skint-1 mAb (10μg/ml) confirmed a consistent decrease in DETC maturation in the presence of the antibody, as judged by the ratio of $V\gamma5^{+}$ $CD45RB^{hi}$: $V\gamma5^{+}$ $CD45RB^{lo}$ cells. The decrease in maturation upon anti-Skint-1 mAb incubation in each experiment was 36% (experiment 1), 59% (experiment 2), and 38% (experiment 3). A paired T test was used to confirm statistical significance ($P = 0.036$).

Supplementary Figure 4. Skint-1 mAb binding to Skint-1 CDR3 loop mutants
Injection of anti-Skint-1 mAb over Skint-1 protein surfaces (wild type Skint-1, 530RU immobilised, blue trace; D127V/D129E Skint-2 loop swop mutant, 525RU immobilised, green trace; D127A/D129A Ala mutant, 516RU immobilised, cyan trace; streptavidin control surface, black trace). Approximately equivalent binding to each surface is detected, indicating the CDR3-like loop of Skint-1 is unlikely to be involved in Skint-1 mAb binding.

Supplementary Figure 5. Dynamics of Skint-1 DV. A. The backbone dynamics are indicated by the order parameter (S^2) of each residue as calculated from the chemical shifts using the $TALOS^{+}$ server using the method described by Berjanskii and Wishart (2). B. Backbone ribbon of the Skint-1 DV, where the width of the ribbon is inversely related to the order. Red is used for regions with greater disorder (S^2 <<1) and white for little disorder (S^2 ~1). Regions highlighted in green could not be assigned and hence no S^2 value could be calculated but presumably the lack of assignments is the result of significant dynamics within these regions.

Supplementary Figure 6. Comparison of Skint-1 DV with structural homologues. A. Overlay of Skint-1 DV (orange) with bovine BTN1A1 (pink; PDB ID 4HH8). B. Overlay of Skint-1 DV (orange) with human BTN3A1 (grey; PDB ID 4F9P; (3)). C. Overlay of Skint-1 DV (orange) with murine MOG (yellow; PDB ID 1PY9;(4)). D. Overlay of Skint-1 DV with human PD-L1 (red; PDB ID 3FN3; (5)).

Supplementary Figure 7. Comparison of Skint-1 DV with MOG and PD-L1. A. (left panel) Interactions at the MOG dimer interface. Secondary structural elements involved in stabilising the MOG dimer interface are highlighted (light blue). (right panel) Putative Skint-1 dimer interface generated using the MOG dimer as a template. The longer C-C’ loop in Skint-1 (light blue) may prevent dimer formation due to major steric clashes with the C-C’ loop of the opposing monomer. B. (left panel) Polar interactions at the PD-L1 dimer interface. Residues that contribute to stabilising the PD-L1 dimer interface are highlighted. Hydrogen bonds are represented by black dashed lines. (right panel) Putative Skint-1 dimer interface generated using PD-L1 dimer as template demonstrates clashes and loss of interactions. Monomers that form each dimer are labelled A and B, respectively.

References

1. Barbee, S. D., Woodward, M.J., Turchinovich, G., Mention, J.J., Lewis, J.M., Boyden, L.M., Lifton, R.P., Tigelaar, R., and Hayday, A.C. (2011) *Proc Natl Acad Sci U S A*. **108**(8):3330-5
2. Berjanskii, M. V., and Wishart, D. S. (2005) *Journal of the American Chemical Society* **127**, 14970-14971
3. Palakodeti, A., Sandstrom, A., Sundaresan, L., Harly, C., Nedellec, S., Olive, D., Scotet, E., Bonneville, M., and Adams, E. J. (2012) *The Journal of biological chemistry* **287**, 32780-32790
4. Clements, C. S., Reid, H. H., Beddoe, T., Tynan, F. E., Perugini, M. A., Johns, T. G., Bernard, C. C., and Rossjohn, J. (2003) *Proceedings of the National Academy of Sciences of the United States of America* **100**, 11059-11064
5. Chen, Y., Liu, P., Gao, F., Cheng, H., Qi, J., and Gao, G. F. (2010) *Protein & cell* **1**, 153-160

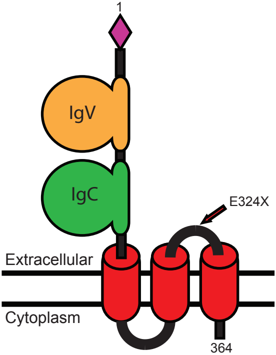

Supplementary Figure 1

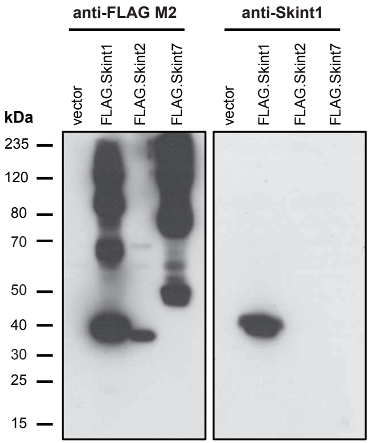

Supplementary Figure 2

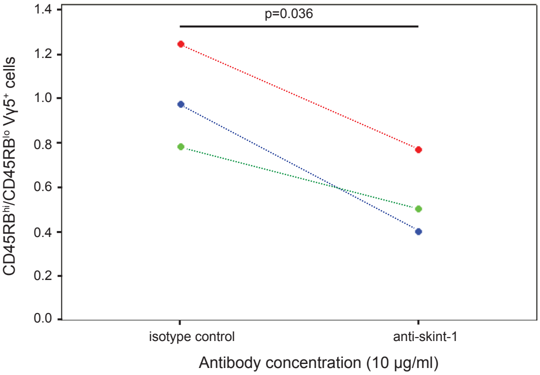

Supplementary Figure 3

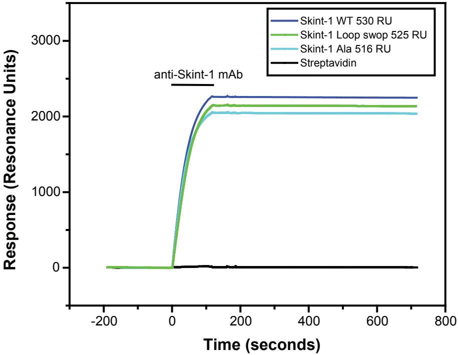

Supplementary Figure 4

A.

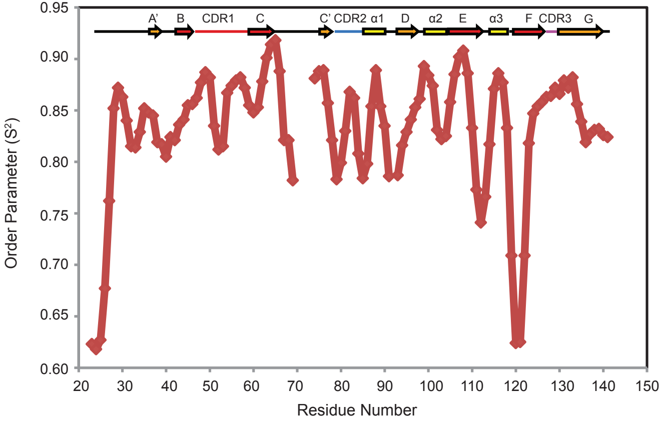

B.

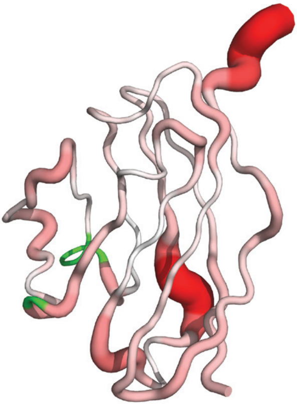

Supplementary Figure 5

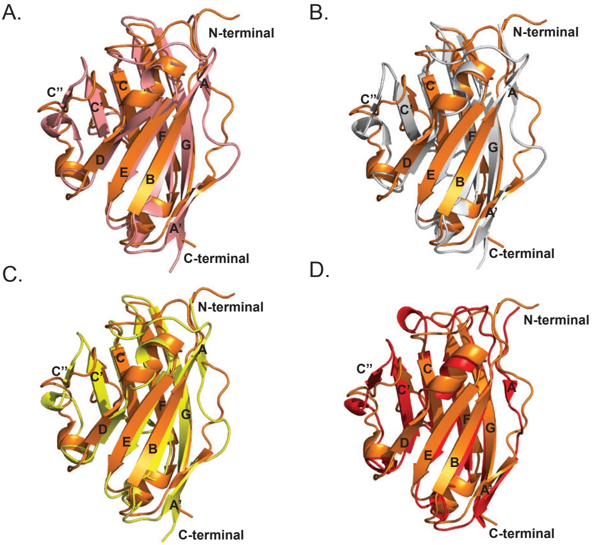

Supplementary Figure 6

A.

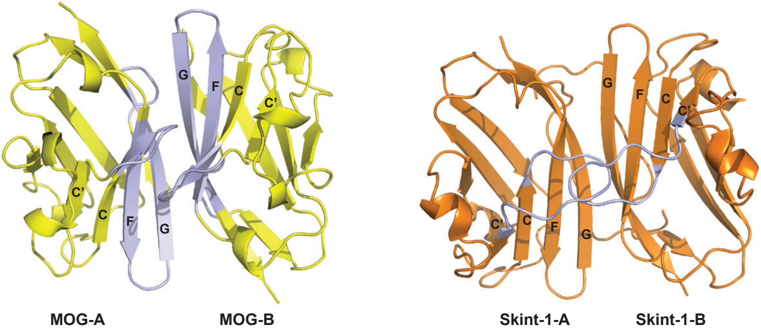

B.

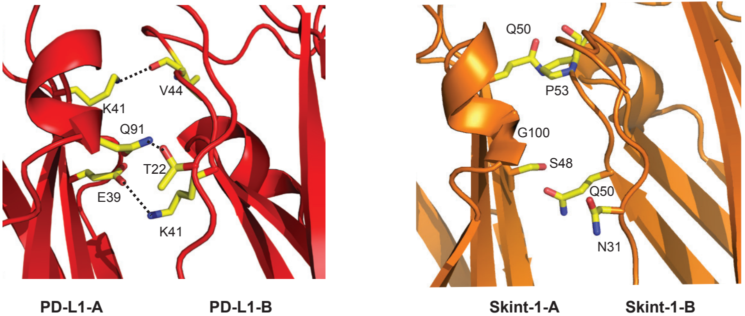

Supplementary Figure 7
